# Minimally invasive surgery versus thoracotomy for locally advanced lung cancers: A systematic review and meta-analysis

**DOI:** 10.1097/MD.0000000000045136

**Published:** 2026-01-16

**Authors:** Yuanfa Chen, Cheng Chen, Dan Ran, Ya Lu, Dong Liu

**Affiliations:** a Department of Thoracic Surgery, Peng Zhou People’s Hospital, Chengdu, Sichuan, China; b Internal Medicine-Cardiovascular Department, Peng Zhou People’s Hospital, Chengdu, Sichuan, China; c Department of Respiratory Medicine, First Affiliated Hospital of Chengdu Medical College, Chengdu, Sichuan, China; d Department of Urology, Peng Zhou People’s Hospital, Chengdu, Sichuan, China

**Keywords:** locally advanced lung cancers, meta-analysis, minimally invasive surgery, thoracotomy

## Abstract

**Background:**

Locally advanced lung cancer is generally regarded as a relative contraindication for minimally invasive surgical approaches. The comparative outcomes between minimally invasive techniques and conventional open thoracotomy in this specific patient population have not been adequately investigated through rigorous clinical studies.

**Methods:**

We systematically searched Embase, Cochrane Library, PubMed, MEDLINE, and Web of Science for studies comparing minimally invasive (robotic and laparoscopic) approaches with conventional open thoracotomy. Pooled standard mean differences, relative risks, and 95% confidence intervals were calculated. The study protocol was registered with PROSPERO (CRD42024526598).

**Results:**

There were 7819 participants including 15 articles. Compared with open thoracotomy, patients undergoing minimally invasive surgery (MIS) demonstrated significantly shorter operative time (SMD = 0.13; 95% CI = 0.01–0.24; *I*^2^=49.9%, *P* = .036) and reduced hospital stay (SMD = −0.19; 95% CI = −0.24 to −0.14; *I*^2^=45.6%, *P* < .001). However, no significant differences were observed in blood loss, ICU days, chest tube duration, lymph node resected, lymph node total stations, complications, acute respiratory distress syndrome (ARDS), atrial arrhythmia, chylothorax, prolonged air leak, pneumonia, and overall recurrence.

**Conclusion:**

MIS demonstrates significant advantages over thoracotomy in terms of operative time and hospital stay for locally advanced lung cancer. However, no statistically significant differences were observed in blood loss, ICU days, chest tube duration, lymph nodes resected, lymph nodes total stations, complications, ARDS, atrial arrhythmia, chylothorax, prolonged air leak, pneumonia, and overall recurrence. More high-quality literature is needed to be included in the research in the future.

## 1. Introduction

The optimal treatment approach for locally advanced non-small cell lung cancer ((LA-NSCLC)) patients have always been controversial. Numerous studies have demonstrated that neoadjuvant chemotherapy combined with surgery provides survival benefits without increasing postoperative complication rates.^[1–3]^ While extensive literature confirms comparable safety and efficacy between minimally invasive surgery (MIS) and conventional thoracotomy for early-to-mid stage lung cancer, with additional advantages in reduced trauma, faster recovery, and improved quality of life.^[4,5]^ Minimally invasive approaches were historically considered contraindicated for LA-NSCLC due to technical challenges and equipment limitations.^[6]^ With technological advancements and refined surgical techniques, thoracic surgeons have progressively expanded minimally invasive applications to LA-NSCLC. Utilizing modern minimally invasive platforms, complex procedures including bronchial sleeve resection, pulmonary artery reconstruction, carinal reconstruction, and combined bronchovascular double-sleeve resections have been successfully performed, continually redefining the boundaries of minimally invasive thoracic surgery.^[7]^

Currently, no meta-analysis has systematically compared MIS with thoracotomy for locally advanced lung cancer. Therefore, we conducted this study to evaluate the comparative efficacy and safety of these 2 surgical approaches for locally advanced lung cancer.

## 2. Methods

### 2.1. Protocol and guidance

The study was performed according to Preferred Reporting Items for Systematic Reviews and the meta-analysis (PRISMA)^[8]^ and the quality evaluation of this article was scored using the Newcastle-Ottawa scale score. The protocols for this review have been registered on PROSPERO (CRD42024526598).

### 2.2. Search strategy

This study involved literature published in the Embase, PubMed, Cochrane Library, Medline, and Web of Science up to January 23, 2024. We defined the eligibility criteria according to the population (P), intervention (I), comparator (C), outcome, and study design approach (O). P: The patients with locally advanced lung cancers. I: undergoing minimally invasive segmentectomy or lobectomy. C: thoracotomy was performed as a comparator. O: one or more of the following outcomes: operation time, blood loss, chest tube duration, ICU days, hospital stay, lymph node resected, lymph node total stations, complications, acute respiratory distress syndrome (ARDS), atrial arrhythmia, chylothorax, prolonged air leak, pneumonia, and overall recurrence.

The MeSH terms “locally advanced lung cancer or lung neoplasm,” “thoracotomy or thoracotomy,” and “robotic surgery or robotic thoracic surgery or video-assisted thoracic surgery or VATS” and comparative study were used. The search strategy was not limited by language or year. It was not requested by the ethics or institutional review committee due to the study being designed as a systematic review and meta-analysis.

### 2.3. Inclusion and exclusion criteria

We have included the literature by the following criteria. Comparative data were available on the treatment of locally advanced lung cancer through minimally invasive and conventional thoracotomy. Outcome indexes should include at least one of the following, perioperative period and oncologic outcomes. Any study which did not confirm the above inclusion criteria was excluded.

### 2.4. Data extraction and outcome measures

Two researchers (CYF and LD) independently reviewed the retrieved literature by the inclusion and exclusion criteria. The third researcher (LY) was asked to participate in the discussion to decide whether to include when disagreements were encountered. The extracted data included the first author, publication, country, study type, group, age, clinical stage, and outcomes (if mentioned) (Table 1).

**Table 1:** The main characteristics of included studies.

Author	Publication	Country	Study period	Study design	Group	Cases	Age^1^	Male (%)^2^	Tumor size^3^	Outcomes	Confounders adjustment	NOS score (max: 9)
Casiraghi et al^[9]^	Journal of clinical medicine	Italy	1998–2022	Retrospective	RAST	16	60–69year: 7	37.5	21 ± 10	*,†,‡,§,‖,¶	Yes (propensity score matching)	8
					Open	16	60–69year: 4	56.3	22 ± 10			
Chen et al^[10]^	J Thorac Cardiovasc Surg	China	since 2010	Prospective	VATS	250	62.2 ± 10.3	62.4	3.3 ± 1.6	†,‡,‖,¶,#,**,††,‡‡,§§,‖‖,¶¶	No	8
					Open	161	60.2 ± 9.6	75.2	4.8 ± 2.2			
Fan et al^[11]^	Journal of thoracic disease	China	2013–2015	Retrospective	VATS	64	59.75 ± 10.5	78.8	4.8 (1–10)	‡,§,¶,#,**,††,¶¶	No	7
					Open	68	59.78 ± 8.4	70.6	5.4 (1.8–10)			
Fang et al^[12]^	J Cardiothorac Surg	China	2013–2017	Retrospective	VATS	14	61 (55–73)	78.6	2.5 (1.0–7.0)	†,‖,¶,**,‡‡	No	7
					Open	67	60 (29–77)	94	3.1 (0.8–8.0)			
Hennon et al^[13]^	Ann Surg Oncol	USA	2002–2007	Retrospective	VATS	95	68.6 (42.4–85.9)	52.6		*,†,‡,§,¶,#,**,‖‖,¶¶	No	8
					Open	19	67.0 (47.8–83.7)	47.4				
Herb et al^[4]^	Seminars in thoracic and cardiovascular surgery	USA	2010–2016	Retrospective	MIS	1862	65 ± 10	48.2		*,‡,**	No	8
					Open	3879	63.8 ± 10	69				
Hireche et al^[14]^	Cancers (Basel)	France	2013–2020	Retrospective	VATS	64	62.03 ± 8.03	56.2	42.3 ± 30	†,‡,‖,¶,#,‡‡,§§,‖‖,¶¶,##	Yes (propensity score matching)	8
					Open	64	63.09 ± 9.9	59.3	31.4 ± 18.9			
Jeon et al^[15]^	Annals of Surgery	Korea	2009–2013	Retrospective	VATS	17	62.7 ± 7.9	82	26.3 ± 12.9	‡,‖‖,‖,¶¶,##	No	8
					Open	18	60 ± 8.7	95	40.6 ± 31.9			
Liu et al^[16]^	Zhongguo Fei Ai Za Zhi	China	2020–2021	Retrospective	RAST	7	61.33 (51–67)	46.14	6.78 (3–13)	*,‖,¶,**	Yes (propensity score matching)	7
					Open	7	62.50 (57–83)	38.46	6.71 (3–15)			
Pan et al^[17]^	Thoracic cancer	China	2016–2021	Retrospective	VATS	14	56.62 ± 10.72	78.57	37.07 ± 8.53	†,§,‖,¶,‡‡,§§,¶¶,##	No	7
					Open	23	56.74 ± 10.36	69.57	39.57 ± 11.49			
Park et al^[5]^	Journal of thoracic disease	USA	2002–2013	Retrospective	MIS	31	67 (50–83)	55		*,†,‡,††,‡‡	No	6
					Open	397	65 (34–87)	46				
Wei et al^[18]^	Annals of surgical oncology	China	2017–2019	Prospective	VATS	63	58.04 (49.18–64.15)	71.43		*,‡,¶	No	8
					Open	54	57.96 (49.49–64.90)	81.48				
Yang et al^[19]^	Eur J Cardiothorac Surg	USA	1996–2012	Retrospective	VATS	30	61.6 ± 11.4	60		‡,‖‖,¶¶	Yes (propensity score matching)	8
					Open	30	60.7 ± 8.9	57				
Zhou et al^[20]^	Thorac Cancer	China	2004–2008	Prospective	VATS	117	58.21 ± 11.00	59		†,¶,#,**,††	No	7
					Open	241	58.64 ± 11.18	64.7				
Zirafa et al^[21]^	Curr Oncol	Italy	2010–2020	Prospective	RAST	61	67.3	62		*,‖	No	8
					Open	70	69.4	73				

Matching: 1 – age; 2 – Male (%); 3 – tumor size.

NA = data not available, NOS score = Newcastle-Ottawa Scale score.

* Chest tube duration.

† Complications.

‡ Hospital stay.

§ ICU, d.

‖ Lymph node resected.

¶ Surgery.

# Atrial arrhythmia.

** Blood loss.

†† Chylothorax.

‡‡ Lymph node total stations.

§§ Overall recurrence.

‖‖ Pneumonia.

¶¶ Prolonged air leak.

## ARDS.

### 2.5. Statistical analysis

Statistical analysis was performed by Stata v.12.0 (Stata Corp LLC, College Station, TX, USA). For this meta-analysis, if the heterogeneity test was *I*^2^ > 50%, *P* < .1, we used the random effect model; if the heterogeneity test was *I*^2^ < 50%, *P* > .1, we used the fixed utility model. The combined *r* values and 95% confidence intervals (CIs) of each study were calculated, and the forest map displayed the characteristics of each study result. The quality of the included literature was evaluated using the Newcastle-Ottawa scale. The Begg and Egger tests were used to test the publication bias. The *P* < .05 was indicated as statistically significant.

## 3. Results

### 3.1. Eligible studies and study characteristics

**Figure 1.**
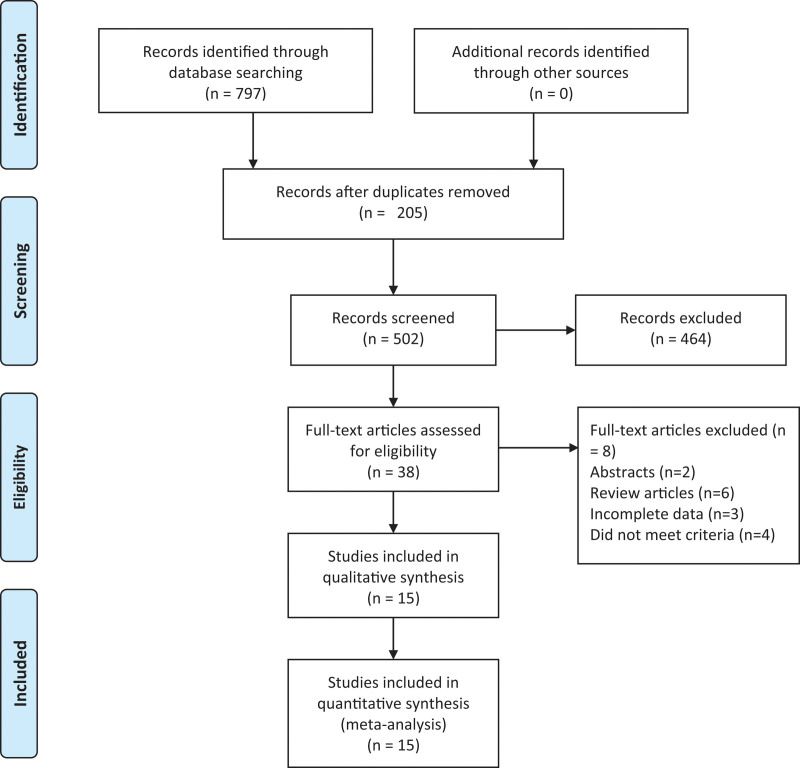
Flowchart for records selection process of the meta-analysis. (According to PRISMA template: Moher D, Liberati A, Tetzlaff J, Altman DG, The PRISMA Group (2009). Preferred Reporting Items for Systematic Reviews and Meta-Analyses: The PRISMA Statement. PLoS Med 6(7): e1000097. doi:10.1371/journal. Pmed 1000097).

We initially screened 797 records. After removing 205 duplicate and cross-published studies, we excluded 464 articles based on title and abstract review. Following a full-text search, reading, and quality assessment of the remaining 38 articles, we ultimately included 15 studies^[4,5,9–21]^ (7819 participants: 2705 in the MIS group vs 5114 in the thoracotomy group) (Fig. 1: PRISMA flow diagram). The detailed characteristics of these studies are presented in Table 1.

### 3.2. Outcomes

#### 3.2.1. Perioperative outcomes

**Figure 2.**
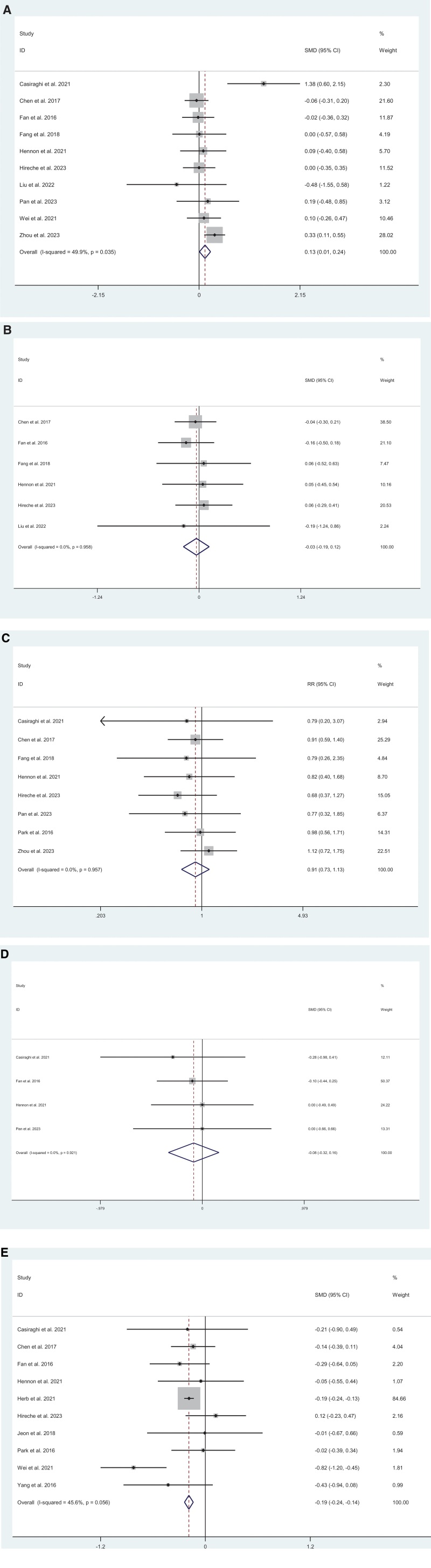
Meta-analysis of minimally invasive surgery versus thoracotomy for locally advanced lung cancers: (A) operation time (*I*^2^ = 49.9%, *P* = .036), (B) blood loss (*I*^2^ = 0%, *P* = .678), (C) chest tube duration (*I*^2^ = 0%, *P* = .374), (D) ICU days (*I*^2^ = 0%, *P* = .506), (E) hospital stay (*I*^2^ = 45.6%, *P* = .000).

Operation time was reported in 10 studies.^[20,16–18,9–14]^ Compared with thoracotomy, MIS was associated with shorter operative time (SMD = −0.13; 95% CI = −0.24 to −0.01; *I*^2^ = 49.9%, *P* = .036) (Fig. 2A). Blood loss was analyzed in 6 studies.^[16,10–14]^ No significant difference was observed between MIS and thoracotomy (SMD = −0.03; 95% CI = −0.19 to 0.12; *I*^2^ = 0%, *P* = .678) (Fig. 2B). Chest tube duration was evaluated in 9 studies.^[20,19,14–17,10–12]^ There was no significant difference between the 2 groups (SMD = 0.91; 95% CI = 0.73–1.13; *I*^2^ = 0%, *P* = .374) (Fig. 2C). ICU stay was reported in 4 studies.^[17,13,11,9]^ No significant difference was found between MIS and thoracotomy (SMD = −0.88; 95% CI = −0.32 to 0.16; *I*^2^ = 0%, *P* = .506) (Fig. 2D). Hospital stay was assessed in 10 studies.^[4,5,19,18,13–15,9–11]^ MIS was associated with a significantly shorter hospital stay compared with thoracotomy (SMD = −0.19; 95% CI = −0.24 to −0.14; *I*^2^ = 45.6%, *P* = .000) (Fig. 2E).

**Figure 3.**
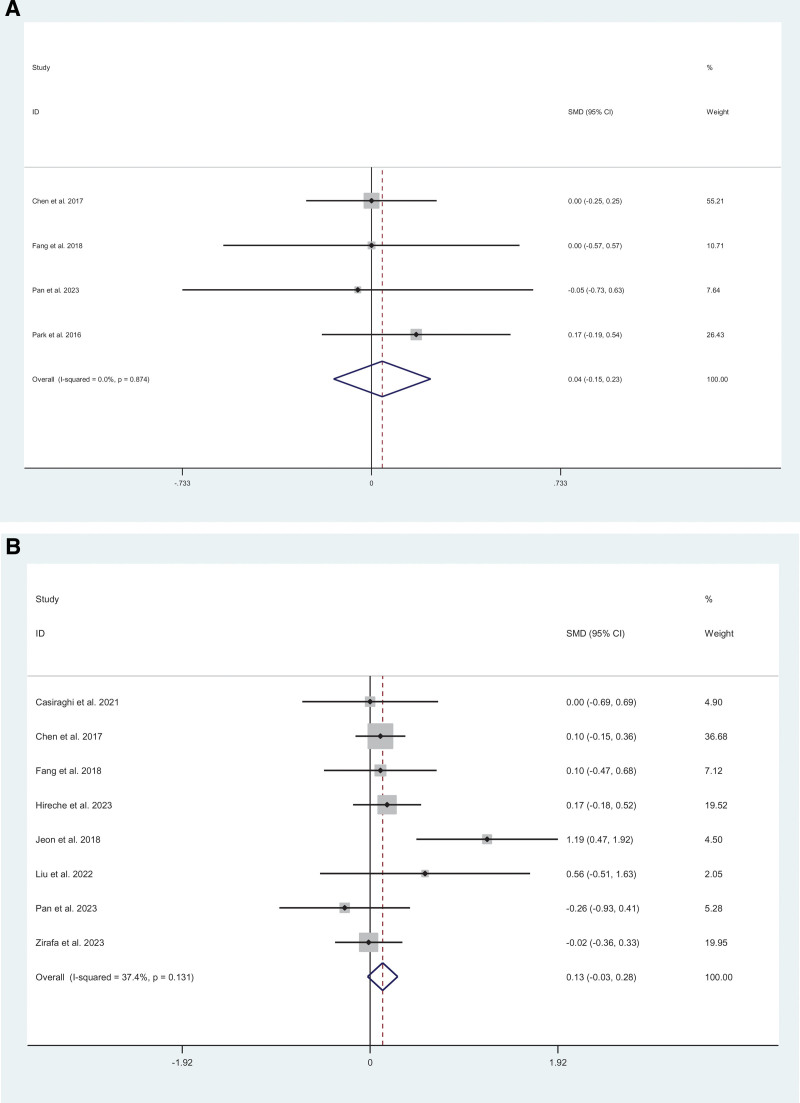
Meta-analysis of minimally invasive surgery versus thoracotomy for locally advanced lung cancers: (A) lymph node total stations (*I*^2^ = 0%, *P* = .663), (B) lymph node total stations (*I*^2^ = 37.4%, *P* = .102).

Lymph node evaluation was reported in 4 studies.^[5,17,12,10]^ No significant difference was found in total lymph node stations between MIS and thoracotomy (SM =  0.04; 95% CI = −0.15 to 0.23; *I*^2^ = 0%, *P* = .663) (Fig. 3A). For lymph node yield, 8 studies were included.^[21,14–17,12,10,9]^ Compared with thoracotomy, patients who underwent MIS had no difference (SMD = 0.13; 95% CI = −0.03 to 0.28; *I*^2^ = 37.4%, *P* = .102) (Fig. 3B).

**Figure 4.**
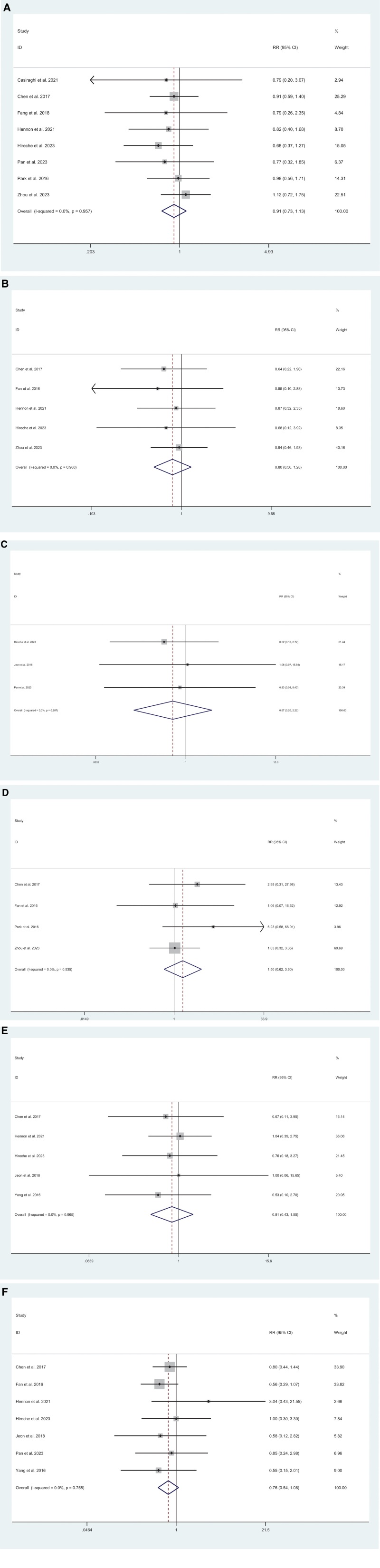
Meta-analysis of minimally invasive surgery versus thoracotomy for locally advanced lung cancers: (A) complications (*I*^2^ = 0%, *P* = .374), (B) atrial arrhythmia (*I*^2^ = 0%, *P* = .346), (C) acute respiratory distress syndrome (ARDS) (*I*^2^ = 0%, *P* = .514), (D) chylothorax (*I*^2^ = 0%, *P* = .368), (E) pneumonia (*I*^2^ = 0%, *P* = .530), (F) prolonged air leak (*I*^2^ = 0%, *P* = .132).

Complications was reported in 4 studies.^[5,20,17,12–14,10,9]^ Compared with thoracotomy, patients who underwent MIS had no difference (RR = 0.91; 95% CI = 0.73–1.13; *I*^2^ = 0%, *P* = .374) (Fig. 4A). We included 5 studies^[20,14,13,11,10]^ about atrial arrhythmia. Compared with thoracotomy, patients who underwent MIS had no difference (RR = 0.80; 95% CI = 0.50–1.28; *I*^2^ = 0%, *P* = .346) (Fig. 4B). Data on ARDS were reported in 3 studies.^[17,15,14]^ Compared with thoracotomy, patients who underwent MIS had no difference (RR = 0.67; 95% CI = 0.20–2.22; *I*^2^ = 0%, *P* = .514) (Fig. 4C). We included 4 studies^[5,20,11,10]^ about chylothorax. Compared with thoracotomy, patients who underwent MIS had no difference (RR = 1.50; 95% CI = 0.62–3.60; *I*^2^ = 0%, *P* = .368) (Fig. 4D). We included 5 studies^[19,13–15,10]^ about pneumonia. Compared with thoracotomy, patients who underwent MIS had no difference (RR = 0.81; 95% CI = 0.43–1.55; *I*^2^ = 0%, *P* = .530) (Fig. 4E). Data on prolonged air leak were reported in 7 studies.^[19,17,13–15,11,10]^ Compared with thoracotomy, patients who underwent MIS had no difference (RR = 0.76; 95% CI = 0.54–1.08; *I*^2^ = 0%, *P* = .132) (Fig. 4F).

#### 3.2.2. Oncology outcomes

**Figure 5.**
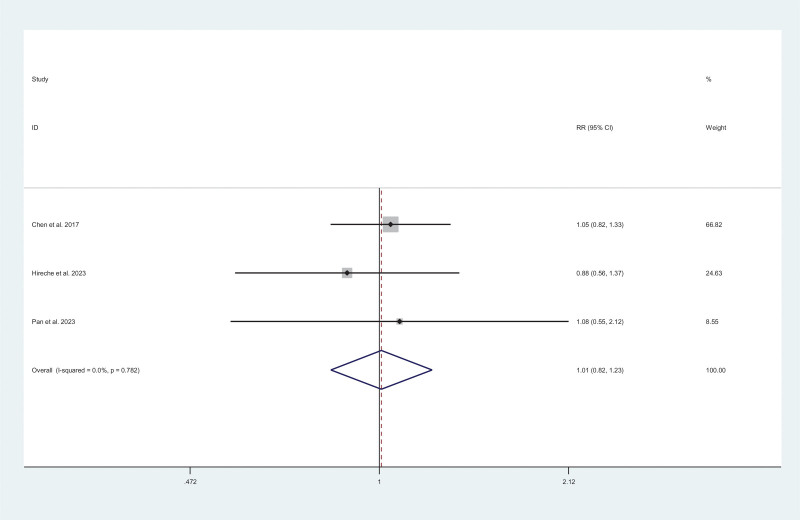
Meta-analysis of minimally invasive surgery versus thoracotomy for locally advanced lung cancers in overall recurrence (*I*^2^ = 0%, *P* = .939).

Overall recurrence was analyzed in 3 studies.^[17,14,10]^ No significant difference was observed between MIS and thoracotomy (RR = 1.01; 95$ CI = 0.82–1.23; *I*^2^ = 0%, *P* = .939) (Fig. 5).

## 4. Publication bias

Publication bias was assessed using Begg test for outcomes with ≥ 10 included studies. For operative time, the Begg test results (*t* = 0.701, *P* = .850; Fig. S1, Supplemental Digital Content, https://links.lww.com/MD/Q325) indicated no significant publication bias.

## 5. Discussion

Compared with conventional open thoracotomy, minimally invasive thoracic surgery is characterized by reduced surgical trauma. This approach utilizes several small incisions combined with video-assisted endoscopic techniques to perform various thoracic procedures. The minimally invasive technique enables complete surgical intervention through these small access ports, resulting in not only shorter treatment duration but also accelerated postoperative recovery.^[22,23]^ For elderly patients with compromised pulmonary function and poor physical condition,^[24]^ or those ineligible for conventional open thoracotomy, MIS provides a valuable alternative. While technically more challenging than traditional open approaches in achieving complete lesion resection with reduced trauma, this method offers distinct advantages including: lower postoperative complication rates, minimized pulmonary function impairment, significantly reduced pain, faster recovery, shorter hospital stays, and decreased healthcare costs.^[4,5]^

Extensive literature has established the completeness and efficacy of minimally invasive approaches for early- to mid-stage lung cancer,^[25,26]^ However, its application in locally advanced disease remains investigational.^[21,17,16]^ With accumulating experience in video-assisted thoracic surgery (VATS) and advancements in surgical instrumentation, previously contraindicated cases – including larger tumors or those with critical structural involvement – have become increasingly feasible for complete thoracoscopic resection.^[4,18,9]^ Akay et al.^[27]^ demonstrated comparable survival outcomes between extrapleural resection and complete chest wall resection for tumors invading (but not extending beyond) the parietal pleura, thereby supporting thoracoscopic approaches for selected cases with limited chest wall invasion.

In this study, patients undergoing MIS demonstrated significantly shorter operative times compared to those receiving open thoracotomy. The conventional open approach was associated with prolonged surgical duration, extended anesthesia time, and greater surgical trauma. This difference primarily stems from the substantial time required for chest opening and closure in open procedures, which consequently increases intraoperative blood loss. In contrast, minimally invasive techniques utilize smaller incisions, significantly reducing chest access time. Furthermore, ongoing advancements in imaging systems and surgical instrumentation are progressively widening this procedural efficiency gap. However, these technical advantages of MIS paradoxically correlate with increased hospitalization duration in our findings.

Lymph node dissection is a critical component of radical lung cancer surgery, and the presence of lymph node metastasis significantly impacts prognosis. Studies have demonstrated that more extensive lymph node dissection correlates with improved survival rates and better long-term outcomes.^[28]^ While multiple investigations^[29,30]^ indicate that lymph node dissection via MIS is non-inferior to conventional thoracotomy, existing literature primarily focuses on early- to mid-stage lung cancer. Nevertheless, minimally invasive techniques offer superior visualization and multi-angular access, suggesting comparable efficacy even for lymph node dissection in locally advanced lung cancer.^[15,14]^ The success of this approach depends on 2 key factors: appropriate patient selection, particularly in cases with mediastinal lymph node enlargement, and the surgeon’s proficiency in advanced minimally invasive techniques. In this study, the scope of lymph node dissection was the same between the minimally invasive group and the conventional open thoracotomy group, and there was no statistical difference in the number of lymph nodes and stations cleaned, which was consistent with the results reported in the literature, confirming that there was no difference in lymph node dissection between the 2 groups.

Studies have shown that the probability of postoperative pulmonary infection, arrhythmia, atelectasis, etc, in patients undergoing MIS is significantly lower than that in patients undergoing conventional open thoracotomy.^[31,32]^ MIS results in minimal muscle damage and mild postoperative pain. Patients can get out of bed and move around early, which is beneficial for deep breathing and active coughing and sputum excretion. At the same time, the surgical field is clear, the surrounding tissue is less damaged, there is less wound exudation, and there is less loss of plasma proteins. In addition, literature review has shown that study had evaluated the impact of thoracoscopic minimally invasive and conventional open chest surgery on the immune status of the body by measuring changes in postoperative IL-10 and C-reactive protein (CRP) concentrations, indicating that MIS has a minimal impact on the immune system.^[32]^ Therefore, minimally invasive surgery is beneficial for the recovery of postoperative body function and the reduction of postoperative complications. In this study, there are no difference in blood loss, ICU days, chest tube duration, lymph node resected, lymph node total stations, complications, ARDS, atrial arrhythmia, chylothorax, prolonged air leak, and pneumonia, consistent with literature, indicating that thoracoscopy does not increase perioperative risk in stage locally advanced lung cancer.

As for overall recurrence, with the increasing improvement of minimally invasive surgery, it is almost no different from conventional open thoracotomy,^[17,14,10]^ and our conclusion also confirms this.

After analyzing the included literature and data, this study has the following limitations: The research subjects of the included literature are mainly from Europe, America, and East Asia, which may bring certain selection biases. The included studies are retrospective studies or prospective cohort studies, which will inevitably be affected by selection bias. In terms of the baseline report of the cases included in the literature, only some of them were provided. Of course, we analyzed the baseline data that can be extracted from the included literature, but we still lacked the comprehensiveness of the data, and could not conduct subgroup analysis according to general characteristics, such as BMI value, In terms of analysis indicators, the long-term efficacy, such as local tumor recurrence rate, was not analyzed by subgroup according to the follow-up time, while only 3 articles were included in the overall recurrence, and the number of articles included in the analysis was insufficient.

## 6. Conclusion

Minimally invasive surgery demonstrates significant advantages over thoracotomy in terms of operative time and hospital stay for locally advanced lung cancer. However, no statistically significant differences were observed in blood loss, ICU days, chest tube duration, lymph nodes resected, lymph nodes total stations, complications, ARDS, atrial arrhythmia, chylothorax, prolonged air leak, pneumonia, and overall recurrence. More high-quality literature is needed to be included in the research in the future.

## Author contributions

**Conceptualization:** Cheng Chen.

**Data curation:** Yuanfa Chen.

**Formal analysis:** Cheng Chen.

**Funding acquisition:** Yuanfa Chen.

**Investigation:** Cheng Chen.

**Methodology:** Yuanfa Chen.

**Project administration:** Cheng Chen.

**Software:** Cheng Chen.

**Supervision:** Dan Ran.

**Validation:** Dan Ran.

**Visualization:** Ya Lu.

**Writing – original draft:** Dong Lin.

**Writing – review & editing:** Dong Lin.

## Supplementary Material


