# Supplementary material for: Minimally invasive surgery versus thoracotomy for locally advanced lung cancers: A systematic review and meta-analysis
Source: Medicine (Baltimore). 2026 Jan 16;105(3):e45136. doi: 10.1097/MD.0000000000045136 (PMC12826292; doi:10.1097/MD.0000000000045136)
Supplement: Supplementary file 1 [file medi-105-e45136-s001.docx]

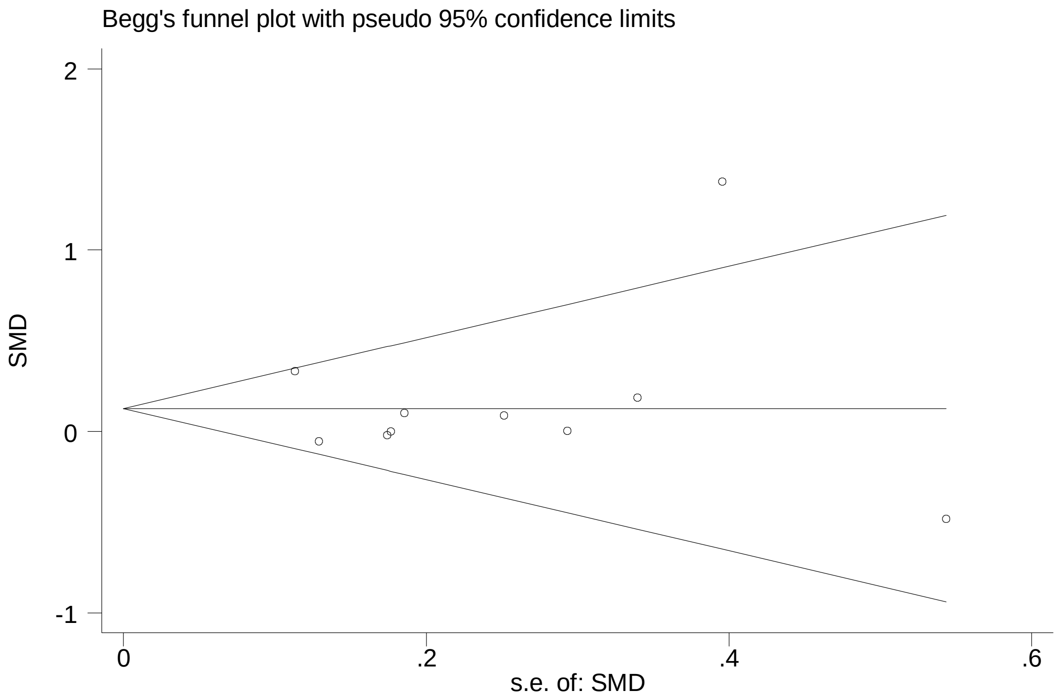
Begg's funnel plot with pseudo 95% confidence limits
2
1
0
-1
SMD
0
.2
.4
.6
s.e. of: SMD
